# Identification of DNA methylation signatures associated with poor outcome in lower-risk Stage, Size, Grade and Necrosis (SSIGN) score clear cell renal cell cancer

**DOI:** 10.1186/s13148-020-00998-z

**Published:** 2021-01-18

**Authors:** Louis Y. El Khoury, Shuang Fu, Ryan A. Hlady, Ryan T. Wagner, Liguo Wang, Jeanette E. Eckel-Passow, Erik P. Castle, Melissa L. Stanton, R. Houston Thompson, Alexander S. Parker, Thai H. Ho, Keith D. Robertson

**Affiliations:** 1 Department of Molecular Pharmacology and Experimental Therapeutics, Mayo Clinic, Rochester, MN USA; 2 Center for Individualized Medicine, Epigenomics Program, Mayo Clinic, 200 First Street SW, Rochester, MN 55905 USA; 3 Hematology Laboratory, Shengjing Hospital of China Medical University, Shenyang, China; 4 Division of Biomedical Statistics and Informatics, Department of Health Science Research, Mayo Clinic, Rochester, MN USA; 5 Department of Urology, Mayo Clinic, Phoenix, AZ USA; 6 Department of Laboratory Medicine and Pathology, Mayo Clinic, Phoenix, AZ USA; 7 Department of Urology, Mayo Clinic, Rochester, MN USA; 8 Office of Research Affairs, University of Florida, Jacksonville, FL USA; 9 Division of Hematology and Medical Oncology, Mayo Clinic, 13400 E. Shea Blvd, Scottsdale, AZ 85259 USA

**Keywords:** Kidney cancer, ccRCC, DNA methylation, Epigenetics, SSIGN score, Prognostic marker, Reduced representation bisulfite sequencing (RRBS), Precision medicine

## Abstract

**Background:**

Despite using prognostic algorithms and standard surveillance guidelines, 17% of patients initially diagnosed with low risk clear cell renal cell carcinoma (ccRCC) ultimately relapse and die of recurrent disease, indicating additional molecular parameters are needed for improved prognosis.

**Results:**

To address the gap in ccRCC prognostication in the lower risk population, we performed a genome-wide analysis for methylation signatures capable of distinguishing recurrent and non-recurrent ccRCCs within the subgroup classified as ‘low risk’ by the Mayo Clinic Stage, Size, Grade, and Necrosis score (SSIGN 0–3). This approach revealed that recurrent patients have globally hypermethylated tumors and differ in methylation significantly at 5929 CpGs. Differentially methylated CpGs (DMCpGs) were enriched in regulatory regions and genes modulating cell growth and invasion. A subset of DMCpGs stratified low SSIGN groups into high and low risk of recurrence in independent data sets, indicating that DNA methylation enhances the prognostic power of the SSIGN score.

**Conclusions:**

This study reports a global DNA hypermethylation in tumors of recurrent ccRCC patients. Furthermore, DMCpGs were capable of discriminating between aggressive and less aggressive tumors, in addition to SSIGN score. Therefore, DNA methylation presents itself as a potentially strong biomarker to further improve prognostic power in patients with low risk SSIGN score (0–3).

## Introduction

Cancers of the kidney and renal pelvis affect > 65,000 patients annually and rank 8th in causes of cancer-related death in the United States. Renal cell carcinoma (RCC) accounts for > 90% of kidney cancers, and the vast majority of RCC tumors (> 80%) are histologically classified as clear cell (ccRCC). Surgical excision by partial or radical nephrectomy remains the standard of care for patients with early stage tumors, however even if surveillance guidelines are followed, ~ 17% of patients with good prognosis still progress to distant metastases after surgery for localized disease [1]. Despite some advances in systemic therapy (e.g. VEGF-targeting treatments), median survival drops to < 19 months after development of metastatic disease [2].

Dysregulation of the von Hippel-Lindau tumor suppressor (*VHL*) gene is nearly universal in ccRCC and typically the initiating event. Subsequent loss of *PBRM1*, *SETD2*, *KDM5C*, and *BAP1* are common secondary events that drive disease progression [3, 4]. CcRCCs manifest among the lowest frequency of structural and copy number variants [5–8]. Additionally, they are on the low end of the frequency spectrum for all types of genetic variation, including many of the classical cancer-associated driver pathways (e.g. *RAS*, *BRAF*, *TP53*, and *RB*) [4, 9]. These observations, along with the frequent mutation of epigenetic regulators [dominated by *SETD2* (2–16%), *PBRM1* (1–43%), *KDM5C* (18%), and *BAP1* (1–17%)] [4], emphasize the importance of epigenetic deregulation to the initiation and progression of ccRCC. This is especially true when coupled with the loss of the *VHL* gene [10], and our previous study showing that *SETD2* mutations drive a DNA hypermethylator phenotype linked to more aggressive clinical features [11]. In addition to the importance of elucidating roles for epigenetic deregulation in understanding the molecular underpinnings of ccRCC, other studies highlight the significant potential of epigenetic modifications as ccRCC prognostic and diagnostic signatures [12, 13]. Our group and others have published how these epigenetic mutations may improve upon prognostic algorithms based on clinicopathological variables [14, 15].

The epigenome is profoundly disrupted in cancer. Epigenetic marks on the DNA, including 5-methylcytosine (5mC) and 5-hydroxymethylcytosine (5hmC), play key roles in development and normal cellular homeostasis [16]. Alterations in 5mC and 5hmC are common events across all cancers, and typically manifest as genome-wide reduction and regional increases in 5mC and 5hmC levels. In particular, losses of 5hmC at active enhancers and gains of 5mC at both enhancers and promoters is linked to downregulation of tumor and metastasis suppressor loci [17, 18]. 5mC is a potent transcriptional repressive signal when present in promoters and enhancers, but is associated positively with transcription when found in gene bodies [19]. In the context of RCC, studies by The Cancer Genome Atlas (TCGA) revealed distinct DNA hypermethylation events linked to poor patient survival and advanced disease stage [17, 20]. Loss of 5hmC and gain in 5mC at kidney enhancers is associated with gene deregulation and poor patient outcome [18, 21]. Collectively, these findings emphasize the importance of epigenetic deregulation in the development of ccRCC. However, much remains to be understood regarding the specific gene targets of epigenetic deregulation that drive the disease process.

The Mayo Clinic Stage, Size, Grade, and Necrosis (SSIGN) scoring system was developed in 2002 due to the poor performance of TNM staging alone to prognosticate risk of death from ccRCC [22]. This algorithm was independently validated [23] and has continued to be useful in the clinical management of ccRCC patients by increasing prognostic accuracy [1]. Even if national guidelines from the NCCN (National Comprehensive Cancer Network) or AUA (American Urological Association) are followed, ~ 17% of patients with “low risk” tumors experience recurrence [1, 24]. Such figures indicate that the SSIGN scoring algorithm would benefit from the addition of novel molecular parameters to further stratify risk and capture patients for which the SSIGN score fails. A number of studies, including those of TCGA, clearly demonstrate the value of integrating molecular parameters like mutational and epigenetic information into traditional histologic methods [3, 17, 25, 26]. In particular there is a need to focus discovery efforts on those ccRCC patients with “low risk” tumors that end up, deceptively, having a greater risk of poor outcome. To address this gap we identified a cohort of ccRCC patients with SSIGN values between 0 and 3 (i.e. predicted median survival of > 25 years [22]), yet died of disease recurrence within a median period of 2.5 years. To identify epigenetic signatures distinguishing the recurrent from the non-recurrent group, we analyzed genome-wide DNA methylation patterns by reduced representation bisulfite sequencing (RRBS) [27] using archival FFPE tissue. We show that recurrent (but low SSIGN score) patients have globally hypermethylated tumors. Furthermore, we discovered 5929 CpGs with significantly different 5mC levels between recurrent and non-recurrent groups (*p* < 0.01 and methylation change |Δ*β*|≥ 10%) that span a number of putative growth regulatory genes including *SLC12A7*, *PRDM16*, and *PTPRN2*. We also identified a set of 43 CpGs that not only distinguish the low SSIGN recurrent from non-recurrent groups, but also segregate aggressive ccRCCs regardless of SSIGN score in TCGA ccRCC datasets. Taken together, our findings suggest that DNA methylation from archival FFPE tissue may not only serve as a robust marker to further stratify SSIGN score, but also point toward key genes and pathways relevant to ccRCC disease severity.

## Results

### Cohort characteristics

Seventeen percent of patients with favorable ccRCC prognosis (SSIGN 0–3) will experience recurrence even if national surveillance guidelines are followed [24]. Thus, we set out to identify a DNA methylation signature that further stratifies long and short term survivors among patients with low SSIGN scores (0–3 range) in two independent cohorts (cohort 1 for marker discovery; cohort 2 for replication). The clinical characteristics of the two cohorts are summarized in Table 1, with detailed clinicopathologic information on each patient listed in Additional file 1: Table S1. By design, there is no statistically significant difference in age, sex distribution, SSIGN score, tumor size or grade between short term survivors (STS; median survival of 2.5 and 3 years for cohorts 1 and 2, respectively) and long term survivors (LTS; median survival of 9.5 and 13 years for cohorts 1 and 2, respectively). Genomic DNA was isolated from FFPE blocks that represented the highest grade portion of the tumors, and subjected to genome-wide DNA methylation analysis using reduced representation bisulfite sequencing (RRBS).

**Table 1:** Summary of patient clinical and pathologic information for cohorts 1 and 2 from Mayo Clinic

	Cohort 1			Cohort 2			Inter-cohort	
	LTS (*n* = 22)	STS (*n* = 14)	*P* value*	LTS (*n* = 30)	STS (*n* = 27)	*P* value*	LTS	STS
Age (years)	68.5	68.1	0.779	67	64	0.728	0.334	0.401
Sex (males)	15 (68.18%)	12 (86%)	0.236	21 (70%)	14 (51.85%)	0.160	0.887	0.033
SSIGN score (0/1/2/3)	2/2/4/14	3/4/2/5	0.567**	6/7/2/15	8/4/2/13	0.905**	0.567**	0.924**
Stage (1/2/3/4)	20/0/2/0	12/0/2/0	0.999**	20/8/1/1	20/7/0/0	0.994**	0.169**	0.284
Tumor size (cm)	5	4.5	0.373	4.75	4.3	0.711	0.911	0.507
Grade (1/2/3/4)	0/6/16/0	0/5/9/0	0.962	2/14/14/0	3/14/10/0	0.985**	0.550	0.663
Necrosis (yes)	3 (13.64%)	1 (7.14%)	0.999***	1 (3.33%)	3 (11.11%)	0.336***	0.299***	1.000***

Values of continuous variables are displayed as median

Inter-cohort section shows the *P* values of the analysis of LTS_cohort 1_ versus LTS_cohort 2_, and STS_cohort 1_ versus STS_cohort 2_

^*^P value of the non-parametric Wilcoxon rank test for continuous data and *χ*^2^ for categorical data

^**^P value resulting from using a Yates *χ*^2^ test

^***^P value resulting from using a 2 × 2 Fisher’s test

### Discovery of differentially methylated CpGs between STS and LTS groups

**Fig. 1:**
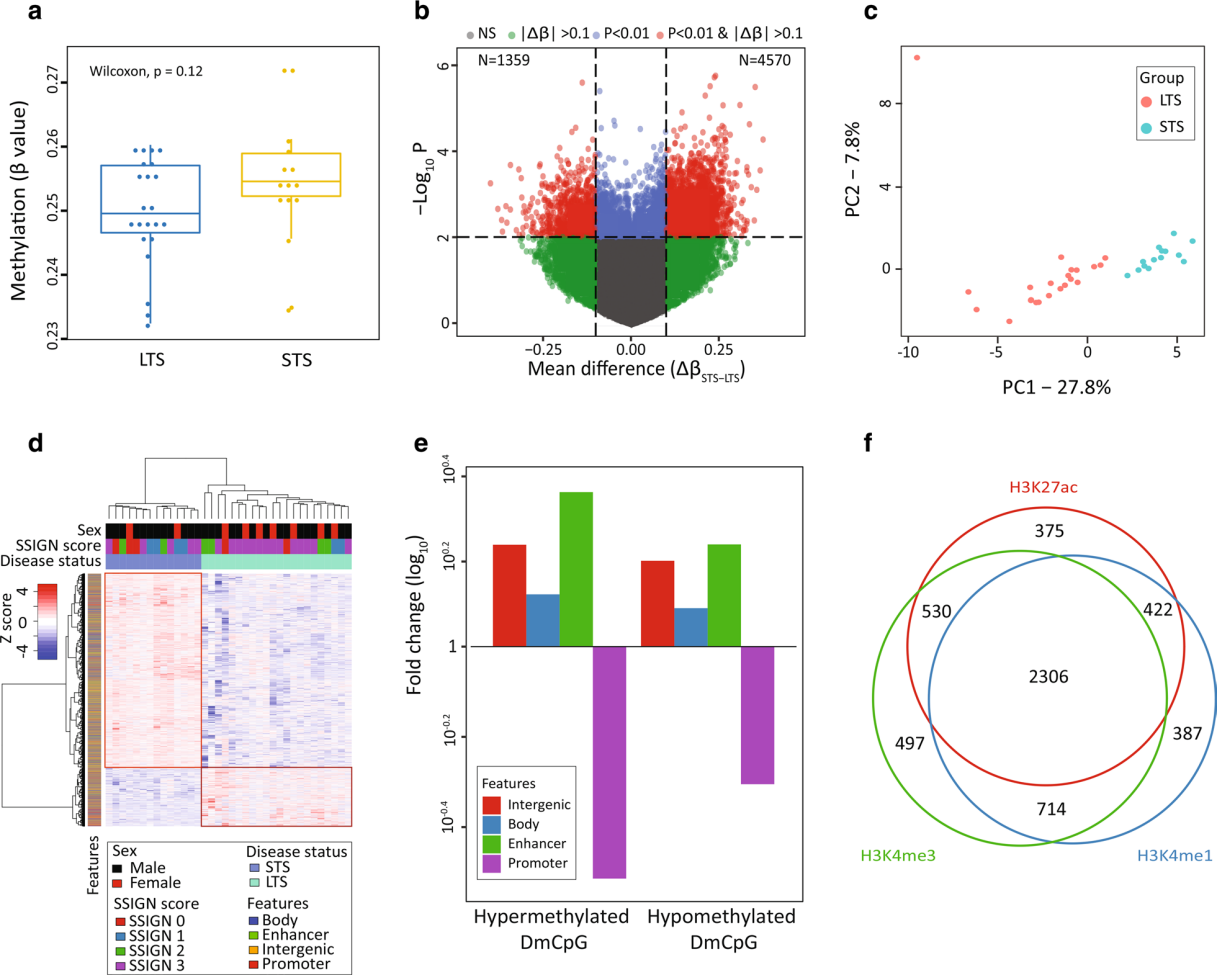
Discovery and characterization of differential methylation characteristic of short term survivors. **a** Boxplot showing the global distribution of methylation for LTS (*n* = 22) and STS (*n* = 14) samples in cohort 1. Y-axis—mean methylation *β* value for each sample. Each dot represents the mean methylation per sample. The non-parametric Wilcoxon test *P* value shows no significant difference between groups, although there is a trend toward hypermethylation in STS tumors. **b** Volcano plot showing differential STS/LTS methylation. X-axis: mean change in methylation (Δ*β*_(STS—LTS)_) for each CpG, y-axis: − log_10_ (*P* value) using a *t* test. The dotted horizontal line represents the *P* value cutoff of 0.01; two dotted vertical lines delineate the Δ*β*_(STS–LTS)_ cutoff set at − 0.1 and 0.1. Each dot represents a CpG: sites in red (*n* = 5929) meet the *P* value and Δ*β* cutoffs, and are thus referred to as differentially methylated CpGs (DMCpGs). **c** PCA plot using the 5929 DMCpG shows separation of pathologic groups into distinct clusters. LTS-red dots, STS-blue dots. The percentage variation between groups explained by each of the principal components is indicated. **d** Heatmap showing supervised hierarchical clustering of LTS and STS using the 5929 DMCpGs reveals two distinct clusters marked by red rectangles: one is hypermethylated (*n* = 4570) and the other hypomethylated (*n* = 1359) in STS. Color bars underneath the column dendrogram represent, from top to bottom: sex, SSIGN score, and disease status. The color bar next to the row dendrogram indicates genomic feature. **e** Barplot showing the relative distribution of 4570 hypermethylated and 1359 hypomethylated DMCpGs normalized to the distribution of all 2.4 M CpGs in cohort 1 over four genomic features (intergenic, enhancer, promoter, and body). The distribution of all features is significantly different between the sites in cohort 1 and the total DMCpGs. Y-axis—fold change (log_10_) of each feature. **f** Venn diagram showing the number of DMCpGs overlapping a selection of three histone marks characteristic of regulatory regions: H3K27ac, H3K4me1, and H3K4me3. *N* = 698 are not represented as they do not overlap with any of the three marks

**Fig. 2:**
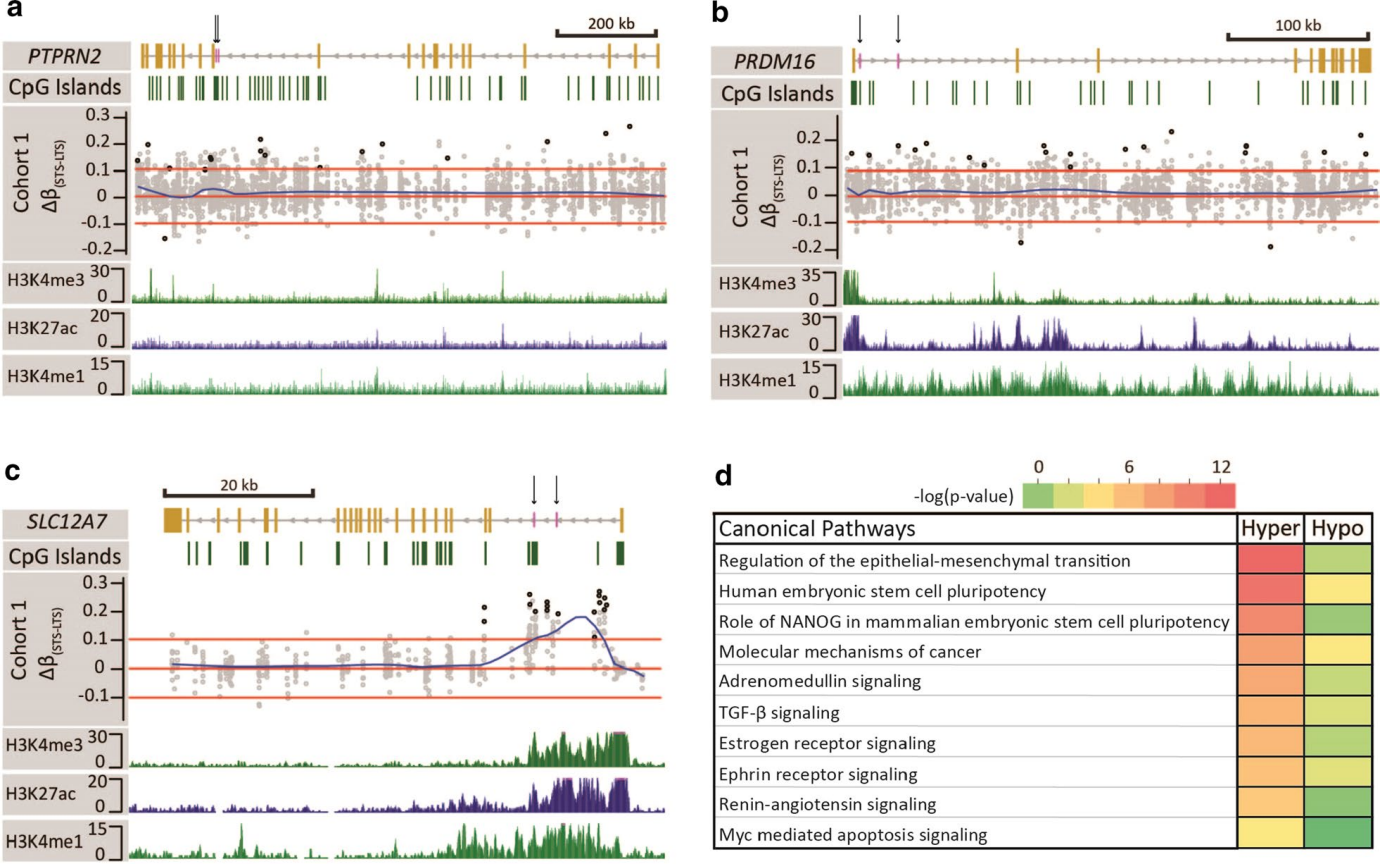
Representative STS/LTS differentially methylated regions and ontology analysis. Browser views of the **a** *PTPRN2*, **b** *PRDM16,* and **c** *SLC12A7* genes. Gene structure is displayed in the first track. Yellow bars represent exons and the grey line introns. Vertical black arrows pointing to short purple bars denote the location of loci that underwent pyrosequencing. Direction of transcription is indicated by arrows on the grey line. The second track displays CpG islands (green bars). Track 3 represent the Δ*β*_(STS—LTS)_ of all CpGs covered by RRBS 5000 bp upstream and downstream of the gene using data from cohort 1. Each grey circle represents a CpG, and the black circles represent the DMCpG meeting * p* < 0.01 and |Δ*β*_(STS–LTS)_|> 0.1 cutoff. Red lines represent the cutoff line at Δ*β*_(STS–LTS)_ ± 0.1. The blue line is a smoothed distribution of the data. Tracks 4–6 display peaks of the histone marks H3K4me3, H3K27ac, and H3K4me1, respectively derived from normal kidney. **d** Heatmap of ontology enrichments for a top group of pathways derived from genes linked to the 5929 DMCpGs between STS and LTS ccRCCs

We obtained DNA methylation data with ≥ 5X coverage on autosomes and present in ≥ 90% of samples for cohorts 1 and 2 for 2,392,937 and 1,153,661, CpGs, respectively. A summary of RRBS data parameters and quality control measures are provided in Additional file 1: Table S1. Overall, the STS patient group trended toward global hypermethylation relative to the LTS group in both cohorts (Fig. 1a, Additional file 2: Fig. S1a). Using a 10% change in methylation (|Δ*β*|≥ 0.1) and *p* < 0.01 between STS and LTS groups in cohort 1 we identified 5929 differentially methylated CpGs (DMCpG) with 4570 sites hypermethylated in STS and 1359 sites hypomethylated in STS, relative to LTS (Fig. 1b). A preponderance of hypermethylated sites in the STS group (4570; 77% of the 5929 DMCpGs), was consistent with the global trends in methylation we observed, and was highly significant overall for this subset of sites (Additional file 2: Fig. S1b). The 5929 DMCpGs segregated the two ccRCC groups using either PCA or supervised hierarchical clustering (Fig. 1c, d). The latter emphasizes the robust hypermethylation that typifies the STS group of low SSIGN score ccRCC patients (Fig. 1d). Unsupervised hierarchical clustering also segregated STS from LTS groups (Additional file 2: Fig. S2). Closer examination of genomic features associated with the 5929 DMCpGs revealed they were significantly over-represented in gene bodies, normal kidney enhancers, and intergenic regions, and under-represented in promoters (Fig. 1e). Furthermore, the majority (*n* = 5231) of DMCpGs overlap with one or more of three histone marks characteristic of gene regulatory regions based on ENCODE data from normal adult kidney, including H3K4me3 ± H3K27ac (active promoter), H3K4me1 only (poised enhancer), and H3K4me1 + H3K27ac (no H3K4me3, active enhancers, Fig. 1f). Many of the identified DMCpGs reside within large, consistently differentially methylated regions of the genome (> 5 DMCpGs within a locus), and pinpoint several high-confidence target genes including *PRDM16, PTPRN2, SLC12A7, MOB2*, *IRX2*, and *MN1* (Fig. 2a–c, Additional file 2: Fig. S3 and Additional file 1: Table S2). Several of these genes are linked to epithelial-to-mesenchymal transition (EMT), which is central to ccRCC pathogenicity [28], including *PRDM16*, *PTPRN2*, and *SLC12A7*. The full list of genes, CpGs, and feature(s) they reside in is summarized in Additional file 1: Table S2. Taken together, RRBS analysis reveals distinct DNA methylation differences between STS and LTS patients, dominated by hypermethylation at gene regulatory regions in STS patient samples that impact genes linked to EMT and cell migration.

We linked the 5929 DMCpGs to their associated gene(s) using the Genomic Regions Enrichment of Annotation Tool (GREAT), which yielded 5744 genes (4046 genes linked to hypermethylated CpGs, 1698 genes linked to hypomethylated CpGs). The full list of ontology and biological processes associated with these genes is listed in Additional file 1: Table S3. This analysis revealed that genes linked to differentially methylated CpGs are associated with phenotypes related to kidney development, including nephron development and tubule formation (Additional file 1: Table S4). Interestingly, these processes have in common regulation by the Iroquois (*IRX1/2/3*) family of homeo-domain transcription factors, critical players in early kidney specification and development [29, 30]. *IRX1* and *IRX2* are differentially methylated between STS and LTS groups (Additional file 1: Table S2). Consistent with identification of individual targeted methylation changes in the STS group, Ingenuity Pathway Analysis (IPA) of the 5744 genes revealed significant enrichment for EMT, while also highlighting type II diabetes mellitus signaling, and renin-angiotensin signaling (Fig. 2d, Additional file 1: Tables S3 and S5). Taken together, these data reveal that the DMCpGs that distinguish STS and LTS patients are enriched in genes relevant to nephrogenic development and tumorigenic pathways, including EMT, which is linked to poor cancer outcome for many tumor types [31].

We subsequently examined cohort 2 (Table 1), an independent cohort which was sequenced separately from cohort 1, at more modest depth to maximize sample number. There were 1,143,262 total CpGs overlapping between cohort 1 and cohort 2. While not statistically significant, STS patients displayed elevated DNA methylation, consistent with results from cohort 1 (Additional file 2: Fig. S1). Applying the same criteria to cohort 2 (|Δ*β*_(STS-LTS)_|≥ 0.1, *p* value < 0.01), yielded 2888 DMCpGs between STS and LTS patients (hypermethylated in STS, *n* = 2186; hypomethylated in STS, *n* = 702). DMCpGs from both cohorts 1 or 2 were enriched at chromosomal peripheries as observed for chromosomes 1, 9, 11, 16, and 17 (Additional file 2: Fig. S4). Overall, 58.9% of CpGs from both cohorts displayed consistent methylation changes between STS and LTS groups (Additional File 2: Figs. S4b and S5). The full list of differentially methylated CpG sites and their associated gene(s) for cohort 2 is provided in Additional file 1: Table S6. Thus, in addition to cohort 2 confirming the global trend toward hypermethylation of the STS group we showed for cohort 1, a number of loci targeted for hypermethylation in the STS ccRCCs were found consistently across both cohorts, including *PTPRN2* and *PRDM16* (Additional file 2: Fig. S3).

To confirm differential methylation among STS and LTS groups identified by RRBS, we utilized bisulfite pyrosequencing. Two regions each from the *PTPRN2*, *PRDM16*, and *SLC12A7* genes were analyzed (Additional file 2: Fig. S6). DNA from 10 STS and 10 LTS samples (cohort 1) was independently bisulfite modified, amplified with locus-specific primers flanking DMCpGs discovered by RRBS, and methylation levels at all CpGs in the amplicon quantified by pyrosequencing. Results for all six loci (ranging from 2 to 9 CpGs in each amplicon) showed that STS samples were overall more methylated than LTS samples, confirming the direction of methylation change identified in the RRBS assay, and further showed that many CpGs adjacent to significant DMCpGs (by RRBS) were also significantly different by pyrosequencing. This sets the stage for expanding the pool of DMCpGs that could be utilized in a screen for identifying STS patients within the low SSIGN score group (Additional file 2: Fig. S6). Taken together, results from an independent Mayo Clinic low SSIGN ccRCC cohort (cohort 2) confirmed overall findings from our discovery cohort (cohort 1) and furthermore, locus-specific pyrosequencing assays confirmed RRBS-based findings and yielded a promising set of DMCpGs that can distinguish STS from LTS ccRCC patients using archival FFPE material.

### Relationship between STS/LTS tumor groups and the normal kidney

**Fig. 3:**
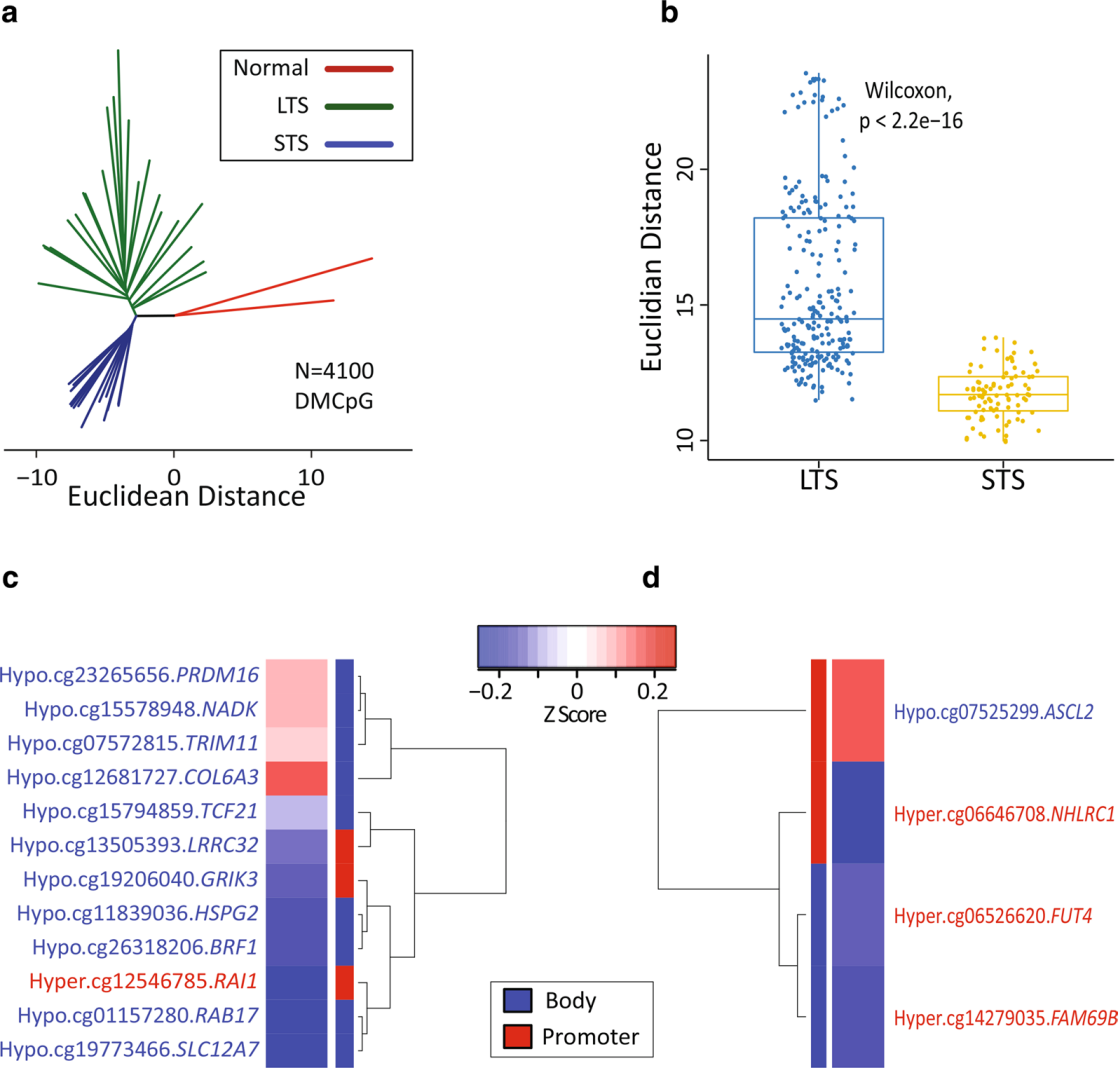
Epigenetic relationships among normal kidney and LTS/STS ccRCC groups. **a** Phyloepigenetic tree derived using 4100 DMCpG shared between the normal kidney samples and the STS/LTS groups. **b** Boxplot comparing the intra-LTS and intra-STS Euclidian distances. Each dot represents the Euclidian distance between any two samples in the group. In the LTS group there are 231 connections, and in the STS group there are 91 connections. **c** Heatmap showing 12/3207 hypermethylated CpGs in cohort 1 significantly associated with survival and significantly correlated with expression of their respective gene, derived from **a**. **d** Heatmap showing 4/893 hypomethylated CpGs in cohort 1 significantly associated with survival and significantly correlated with the expression of their respective gene derived from **a**. The red color in the heatmap represents a positive correlation between the methylation status of the CpG and expression of the respective gene. The blue color in the heatmap represents a negative correlation between methylation status of the CpG and expression level of its respective gene. The color bar next to the dendrogram indicates the genomic feature where each site in located: body (blue), promoter (red). The information on the opposite side of the dendrogram indicates the methylation status linked to longer patient survival (hyper or hypo) for the CpG in KIRC, the GC number of each CpG, and the respective gene name as per Illumina 450K manifest. CpGs in red text are associated with better survival when hypermethylated. CpGs in blue text are associated with better patient survival if hypomethylated

The analyses reported above were focused on identifying epigenetic differences that further stratify STS and LTS amongst patients predicted to be low risk from the Mayo SSIGN score. It is also of interest to examine the relationship between each of these groups and normal kidney DNA methylation patterns to determine whether STS may evolve from LTS tumors or derive independently. RRBS from two normal adult kidneys from individuals without cancer was generated and yielded methylation data on 1,729,885 autosomal CpG sites between the two samples (Additional file 1: Table S1). Of the 5929 DMCpGs between STS and LTS, 4100 were covered in the two normal kidney RRBS samples. To examine methylation levels at these 4100 overlapping CpGs, we performed a phyloepigenetic analysis, which showed that normal kidney occupied a space independent from STS and LTS, while the two low SSIGN groups diverged away from the branch of normal kidney (Fig. 3a). This suggests that short term survivors are not a progression from the LTS epigenome, but rather are independently evolved. In addition, it was observed that the STS tumors were more similar to each other (that is, more closely clustered together) compared to LTS tumors (Fig. 3b). This may be because events driving these clusters are targeted disruptions of the epigenome (to key regulatory regions like promoters and enhancers) rather than the relatively non-specific global hypomethylation observed across most cancer epigenomes.

We further analyzed these 4100 DMCpGs by interfacing them with survival data using DNA methylation data generated by TCGA’s clear cell renal cell cancer project (KIRC). Of the 4100 DMCpGs (3207 hypermethylated; 893 hypomethylated) used in the phyloepigenetic analysis, 132 overlap with TCGA KIRC 450k CpGs, and 43 map to genes. Of these 43 CpGs, 12 from the STS hypermethylated and four from the STS hypomethylated showed significant associations (*p* < 0.05) with ccRCC patient survival and were correlated with expression of their associated gene (Fig. 3c, d). Notably, these included CpGs from *PRDM16* and *SLC12A7*, which were among the highly STS-LTS differentially methylated loci discussed earlier. Taken together, these findings suggest that STS and LTS both evolve from normal kidney and then diverge, and that these differences in DNA methylation have a differential impact on progression at a gene level in STS versus LTS. In other words, the STS group is not evolved from LTS-like tumors, but rather STS represents a distinct epigenetic etiology.

### Identifying CpGs at the extremes of differential methylation across STS and LTS tumor groups

**Fig. 4:**
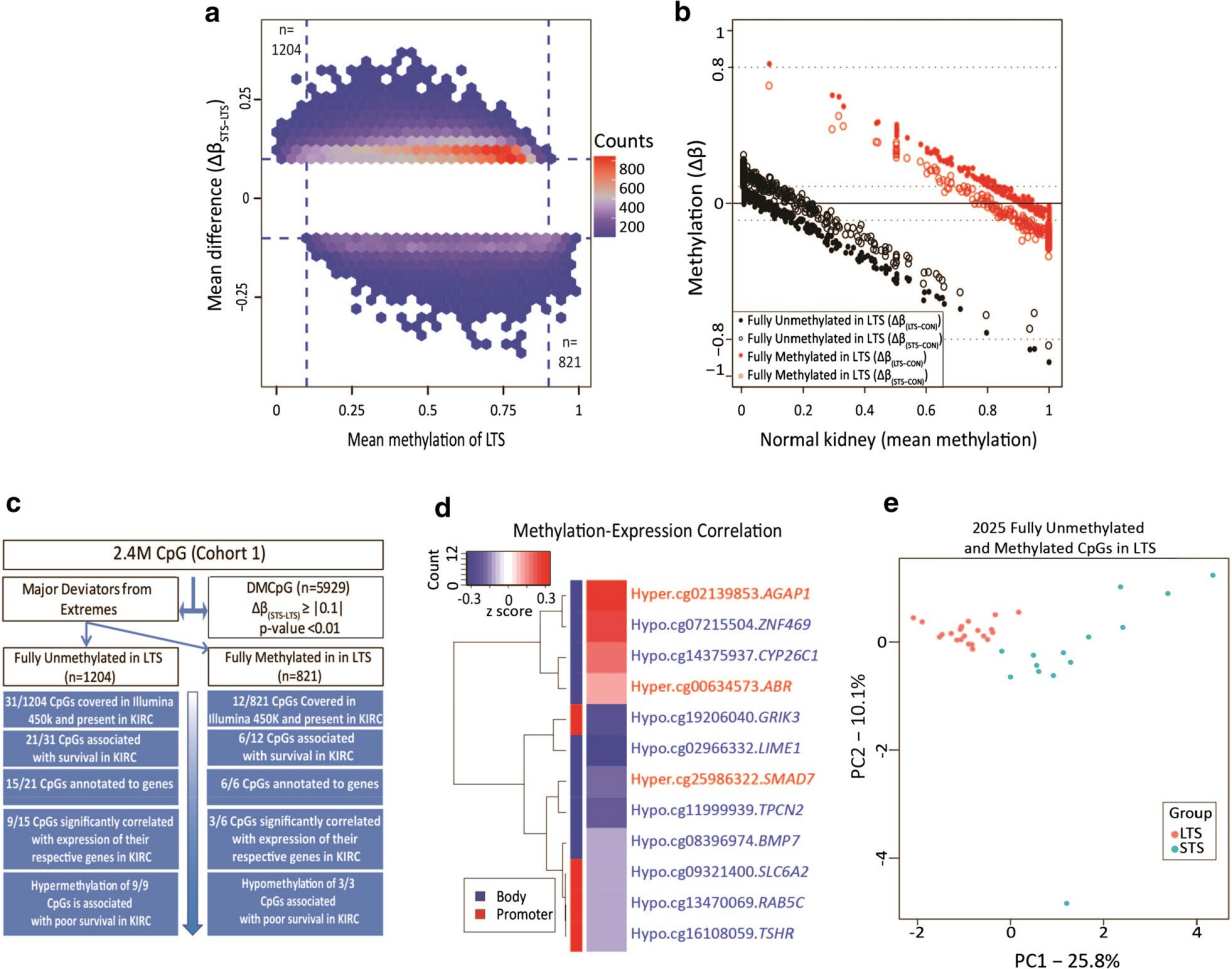
Discovery and characterization of extreme phenotype CpGs among STS and LTS groups. **a** Hexbin plot of the mean methylation of each CpG for the LTS group versus Δ*β*_(STS-LTS)_. Upper left and bottom right regions demarcated by dashed lines indicate CpGs deviating from the extremes in STS and analyzed further. The top-left represents CpGs that are lowly methylated in LTS (*β* = 0 to 0.1; *n* = 1204) and gain a minimum of 10% methylation value in STS. The bottom-right represents CpGs highly methylated in LTS (*β* = 0.9 to 1; *n* = 821) that lose at least 10% methylation in STS. **b** Scatterplot showing methylation changes of LTS (open circles) and STS (solid circles) against normal kidney (CON) plotted against normal kidney methylation levels on the x-axis. Unmethylated CpGs in LTS are colored in black (*n* = 701) and fully methylated CpGs in LTS are represented in red (*n* = 514). **c** Flowchart denoting relationships between the 5929 DMCpGs (from Fig. 1), and the 2025 CpGs deviating from the extremes analyzed here. The overlap of the LTS fully methylated and unmethylated CpGs with TCGA-KIRC is shown in the blue boxes. The progressive analysis of the overlapping CpGs for survival, and methylation-expression correlation are indicated in the flow diagram. This analysis was conducted using 318 KIRC ccRCCs for which both 450K and RNA-seq data were available. **d** Heatmap showing the nine hypermethylated and three hypomethylated CpGs significantly associated with survival and correlated with expression of their respective genes from panel c. Red color in the heatmap represents a positive correlation between the methylation of the CpG and expression of its respective gene; blue color represents a negative correlation. The color bar on the left of the heatmap indicates the genomic feature where each site in located: body (blue), promoter (red). All promoter CpGs are inversely correlated with the expression of their respective genes. The information on the right indicates the methylation status linked to longer patient survival (hyper or hypo) for the CpG in KIRC, the identifier for each CpG, and the name of the associated gene (per Illumina 450K manifest). CpGs in red text are associated with better survival when hypermethylated. CpGs in blue text are associated with better patient survival if hypomethylated. **e** PCA plot of 2025 CpG deviating from extremes in STS showing the ability of these sites to separate LTS (red) and STS (blue) groups. Percentages of variance that can be explained by each principal component are indicated

In a parallel approach, we hypothesized that methylation changes at the extremes of the range, that is hypermethylation at CpGs in STS tumors that have no/low methylation in the LTS group (termed LTS fully unmethylated sites) and hypomethylation of CpGs in STS tumors that are highly methylated in LTS ccRCCs (termed LTS fully methylated sites, Fig. 4), might facilitate identification of functionally relevant epigenetic alterations and changes that are robust markers of STS patients. We therefore plotted mean methylation of CpGs in the LTS group (cohort 1) against the Δ*β*_(STS-LTS)_ at those sites. The LTS fully unmethylated category (≥ 10% gain in the STS tumors, upper left section of Fig. 4a) yielded 1204 CpGs, while the LTS fully methylated category yielded 821 CpG sites (≥ 10% loss of methylation in the STS tumors, lower right section of Fig. 4a). We sought to understand and define baseline methylation of the LTS unmethylated/fully methylated CpG set in normal kidney. As shown in Fig. 4b, the majority of LTS unmethylated CpGs that become hypermethylated in STS patients are lowly methylated in normal kidney, indicating that these are true de novo gains in methylation unique to STS patients. On the other hand, the LTS hypomethylated CpGs (red dots/circles) are more highly methylated in normal kidney, and hypomethylated in both LTS and STS ccRCC, however this hypomethylation was more extensive in STS patients. As locus-specific hypermethylation and genome-wide stochastic hypomethylation events typically characterize cancer cell epigenomes, both of these trends are consistent with STS being epigenetically more ‘progressed’ relative to LTS.

To examine relationships between these differential methylation events and patient survival in an independent cohort, we queried the overlap between our 1204/821 most hyper-/hypomethylated sites among STS and LTS tumors, and TCGA-KIRC, which included 343 ccRCCs with matched DNA methylation and gene expression data. Although the overlap at the single CpG level is limited between RRBS and the Illumina 450k array, we nonetheless identified 49 CpGs in common of which 43 were included in our analysis as they were covered by 90% of samples in KIRC (Additional file 1: Table S7). Of these 43 CpGs, 31 and 12 were present in the LTS fully unmethylated/fully methylated categories, respectively (Fig. 4c). We observed that high methylation levels in 21 out of the 31 CpGs (67.8%) at the LTS unmethylated sites were associated with poor patient survival, consistent with the overall hypermethylation phenotype in short-term survivors. Moreover, these 21 CpGs mapped to 15 genes, and of these 15, approximately two thirds (*n* = 9, 60%) were significantly correlated (*p* < 0.05) with expression of their associated gene, using matched TCGA RNA-seq data (Fig. 4c, d), suggesting that these CpGs functionally contribute to gene expression. Further support of this link is shown in Additional file 2: Fig. S3, where 450k array CpGs in the vicinity of DMCpGs derived from cohorts 1 and 2 were linked to survival using data from TCGA-KIRC. The ‘HR KIRC’ track in each browser view shows the hazard ratio (HR) of each CpG contained in the window of interest and surveyed by the Illumina 450k array. CpGs with HR > 1 are associated with worse survival when hypermethylated. This is concordant with our findings indicating poor survival being associated with hypermethylation since hypermethylation at these sites is observed in the STS group. On the other hand, examination of the LTS fully methylated category yielded 821 CpGs (Fig. 4c). Hypermethylation at six of these sites was associated with better survival in the KIRC dataset, and of these, three were significantly correlated with expression of their respective genes (Fig. 4c, d). Taken together, these findings show that CpGs deviating away from methylation extremes are capable of discriminating between the STS and LTS cancer groups (Fig. 4e; Additional file 2: Fig. S7).

### Extrapolating STS-LTS methylation signatures to public datasets

**Fig. 5:**
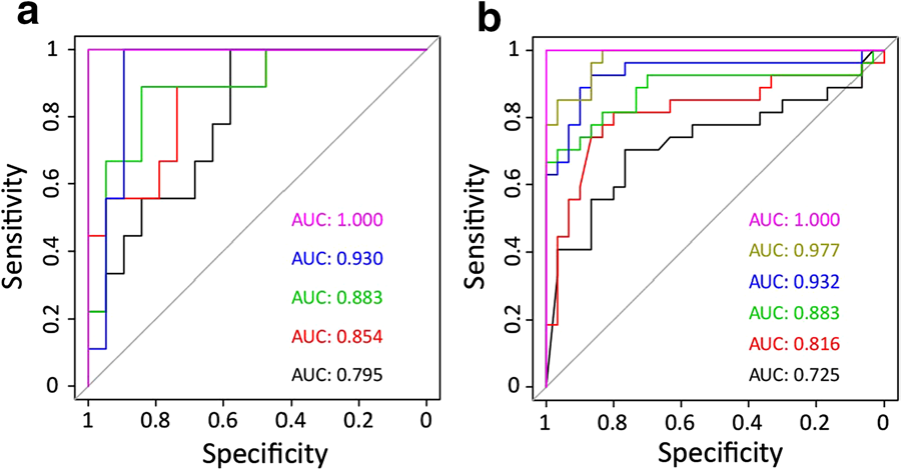
AUROC analysis for CpGs involved with differentiating low SSIGN score ccRCC patients. **a** AUROC using the top five CpGs obtained from a stepwise recursive partitioning analysis performed on the TCGA-KIRC derived LTS-STS cohort (LTS = 19, STS = 9). Each AUC is the cumulative score of the inclusion of an additional CpG in the following order: cg24304972 (black), cg16108059 (red), cg14010015 (green), cg02966332 (blue), and cg02139853 (fuchsia). **b** AUROC using the top six CpGs obtained from a stepwise recursive partitioning analysis performed on the cohort 2 (LTS = 30, STS = 27). Each AUC is the cumulative score of the inclusion of an additional CpG in the following order: chr3:195489634 (black), chr9:137024923 (red), chr5:3591951 (green), chr12:52708570 (blue), chr5:80256046 (kaki), and chr12:129338403 (fuchsia)

To drill deeper into the potential utility of the 2025 extreme change CpGs (1204/821 most hyper-/hypomethylated), we calculated SSIGN scores for all samples in the KIRC dataset using pathologic data provided, then selected samples with SSIGN score ≤ 3 and further stratified by patient survival, as we did for our Mayo Clinic cohorts but with relaxed inclusion criteria to maximize sample number [STS: death in < 7.5 years (SSIGN 0–1) or < 4.5 years (SSIGN 2–3), and LTS: survival > 8.5 years (SSIGN 0–1) or > 5.5 years (SSIGN 2–3)]. This resulted in 29 samples (LTS = 19, STS = 9) in the SSIGN 0–3 range with survival data consistent with our metrics (note that this TCGA-derived case-cohort is not reflective of typical SSIGN 0–3 survival outcomes, but is driven by our specific inclusion criteria). Using this independent cohort, we conducted stepwise recursive partitioning and found that as few as 5/43 CpGs in common between TCGA-KIRC and cohort 1 RRBS (cg24304972, cg16108059, cg14010015, cg02966332, and cg02139853) segregated LTS from STS with an AUC = 1.000 (Fig. 5a). Furthermore, stepwise recursive partitioning using another set of 6/564 CpGs in common between cohort 2 and cohort 1 (chr3:195489634, chr9:137024923, chr5:3591951, chr12:52708570, chr5:80256046, and chr12:129338403) also segregated STS from LTS in cohort 2 with an AUC = 1.000 (Fig. 5b).

**Fig. 6:**
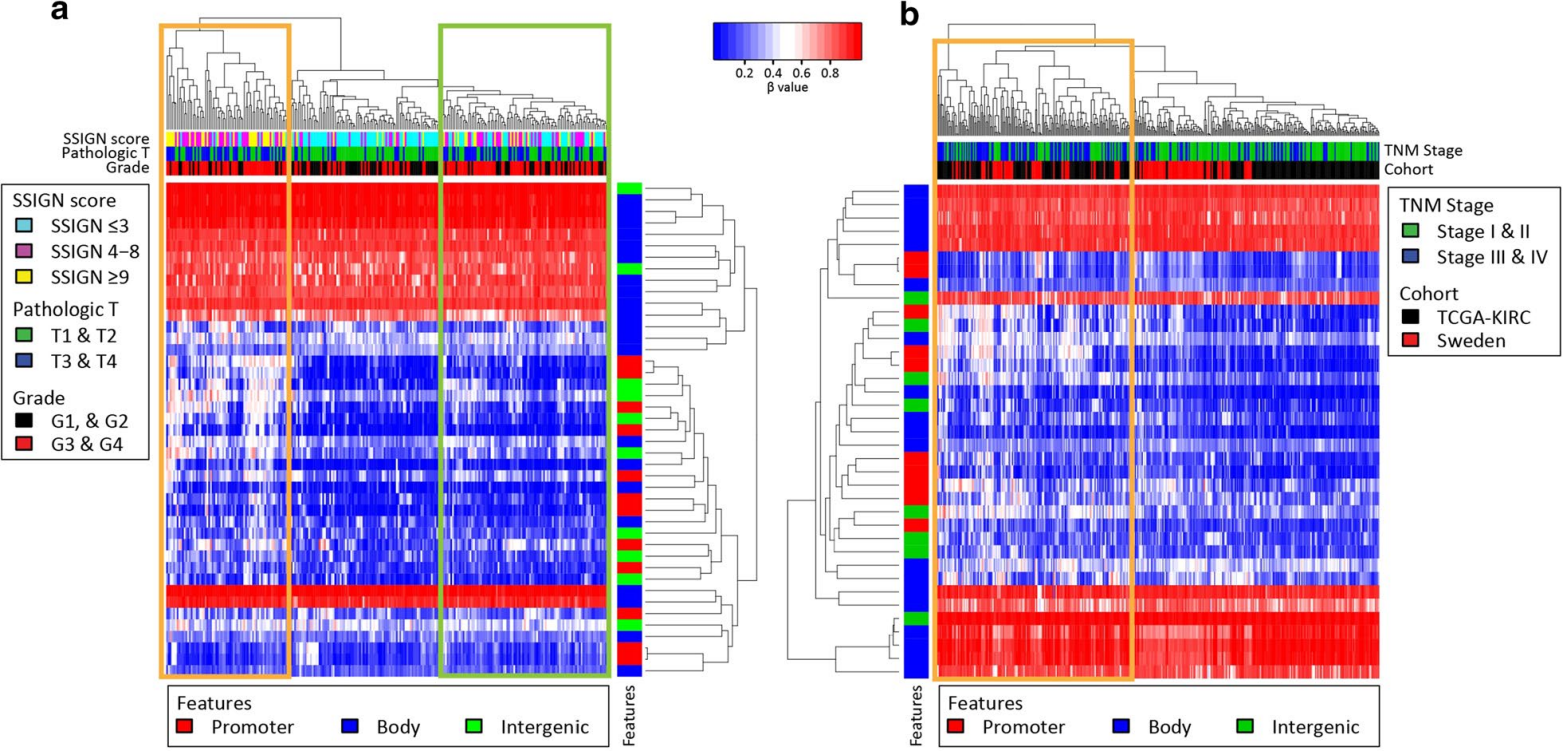
Heatmap and hierarchical clustering of ‘all-comer’ ccRCC samples using a subset of extreme change CpGs. **a** Heatmap showing supervised hierarchical clustering of KIRC ccRCC samples (*n* = 252) driven by methylation beta values at the 43 CpGs from analysis presented in Fig. 4c. Color bars beneath the column dendrogram represent, from top to bottom: SSIGN score, pathologic T stage, and cancer grade. SSIGN scores are categorically divided into 3 groups: ≤ 3, 4–8, and ≥ 9. Pathologic T stages are divided into low (T1 and T2) and high (T3 and T4) stages. Cancer grades are divided into low (G1, and G2), and high (G3 and G4). The color bar next to the row dendrogram indicates the genomic feature. In panel (**a**), when examining 43 CpGs, poor outcome tumors form two clusters: hypermethylated (orange box) and intermediately methylated (green box), which show a significant over representation (*p* < 0.01) of more aggressive tumors (higher SSIGN scores, more aggressive pathologic T status, and cancer grade). **b** Heatmap showing supervised hierarchical clustering of a combination of KIRC (*n* = 252) and the Swedish cohort (*n* = 132) ccRCC samples driven by methylation beta values at the 37/43 CpGs from analysis presented in Fig. 4c. Color bars beneath the column dendrogram represent, from top to bottom: TNM stage, and cohort from which the sample originates. TNM stages are divided into low (I and II) and high (III, IV) stages. The color bar next to the row dendrogram indicates the genomic features. When examining 37 CpGs, more aggressive tumors cluster into one distinct group (orange box)

When the 43 CpGs were examined in all tumors in KIRC where SSIGN score could be calculated (*n* = 252), we observed the formation of three clusters of tumor samples (hypermethylated, hypomethylated, and intermediate) that had significantly different median SSIGN scores (median_hyper_ = 7, median_hypo_ = 2, median_intermediate_ = 5; *p* < 0.001). While we identified these 43 CpGs by comparing STS and LTS patients with low SSIGN score, the DNA methylation signature derived from this more restrictive comparison was capable of stratifying ‘all-comer’ ccRCCs into low, intermediate, and high risk groups. This finding suggests that these CpGs may be central to the pathogenesis of ccRCC, especially when the enrichment of survival-associated genes linked to these CpGs (27/43 CpGs linked to poor prognosis in STS from KIRC data) is also taken into account. Furthermore, the hypermethylated and the intermediate groups contained an over-representation of tumors with high stage and grade (Fig. 6a, orange and green boxes respectively). To identify CpGs significantly associated with SSIGN score, and determine whether a smaller set of CpGs could identify more aggressive KIRC tumors, we performed correlation analysis and selected CpGs that significantly correlated with SSIGN score. This analysis identified 22 CpGs significantly correlated with SSIGN score capable of segregating the most aggressive ccRCCs into a distinct cluster (*p* < 0.01, Additional file 2: Fig. S8, orange box). Consistent with this, the median SSIGN score in the more aggressive and hypermethylated cluster of tumors is significantly higher than that in the less aggressive cluster (median_hyper_ = 7, median_hypo_ = 2; *p* < 0.001). We sought to further increase the size of this cohort by combining it with additional ccRCC samples from a Swedish RCC cohort available from NCBI GEO (GSE113501) [32]. Due to the limited clinical data available for this cohort it was not possible to calculate SSIGN score, therefore we used TNM staging for comparison. Examination of the combined dataset (KIRC and the Swedish cohort), revealed that use of 37/43 CpGs linked to poor prognosis in STS from the RRBS/KIRC overlap set, significantly (*p* < 0.001) separated stage I/II tumors (hypomethylated) from stage III/IV tumors (hypermethylated, Fig. 6b). These findings suggest that the 43 CpGs are capable of segregating ccRCCs based on disease aggressiveness and poor clinical outcome in a larger multicenter independent cohort of patients. Importantly, higher methylation was associated with more aggressive clinical phenotype (e.g. SSIGN score, tumor stage) in a broader patient group than our Mayo Clinic cohorts (TCGA, Swedish cohort), consistent with the fact that they were originally identified in epigenetically more aggressive short-term survivor SSIGN 0–3 tumors.

## Discussion

In the current manuscript we address a significant desideratum in ccRCC patient management to better understand the molecular underpinnings of aggressive and recurrent disease and identify patients with high risk tumors that the current SSIGN score algorithm fails to capture. In other words, what molecular characteristics, specifically DNA methylation signatures, could contribute to tumor aggressiveness independent of currently implemented scoring criteria, with the ultimate goal of better stratifying survival of ccRCC patients? Using genome-wide DNA methylation data derived from RRBS in two ccRCC cohorts from Mayo Clinic, we identified a set of CpGs that show robust differences in methylation between the STS and LTS groups. Methylation at a subset of these CpG sites is capable of segregating aggressive from less aggressive tumors in 450k-derived DNA methylation data from TCGA-KIRC and an independent Swedish cohort. In addition, locus-specific pyrosequencing shows that key STS/LTS methylation differences are reproducible at individual CpGs using an independent assay method. With as few as five CpGs, we achieve > 95% sensitivity and specificity in distinguishing between STS and LTS patients within the lower risk (0–3) SSIGN score category. This suggests that the CpGs identified here represent promising epigenetic biomarkers that should be further validated in larger cohorts. Our study is limited in that our cohorts are relatively small and from a single institution, and it will be critical to validate the markers we identified in larger independent cohorts of SSIGN score 0–3 tumors. We intentionally selected lower risk SSIGN 0–3 tumors because of the longer overall survival of these patients where a misclassification would lead to a higher number of years of lost life (median survival of cohort 1 LTS was 9.5 years whereas it was 2.5 years for the STS group). While promising as high-risk biomarkers, genes associated with the differentially methylated regions are enriched in processes such as EMT, VEGF and TGF-beta signaling, stemness, and cancer/metastasis signaling pathways, suggesting that these epigenetic changes functionally drive disease aggressiveness. The globally elevated level of DNA methylation in high risk STS patients may further serve as a marker for this group of patients by exploiting immunohistochemical stains for 5-methylcytosine or mass spectrometry-based quantification. Taken together, our study reveals novel epigenetic differences characteristic of high risk ccRCCs that are paradoxically classified as lower risk by the SSIGN score. To better identify patients in this group, and to provide more personalized treatments, the newly discovered epigenetic markers should be further researched and understood. It is encouraging to note that our methylation signature obtained from FFPE blocks should be readily amenable to clinical implementation since this is the most widely used method of tissue preservation.

Prognostic models for metastatic RCC, such as the International Metastatic RCC Database Consortium Risk Model [33] and the Memorial Sloan-Kettering Cancer Center Score for Metastatic RCC [34], have been previously described and attempts were made to incorporate genomic information into furthering predictions [35]. However, unlike these models, the Mayo Clinic SSIGN score is specific for localized ccRCC. In fact, the SSIGN score correctly predicts larger, aggressive tumors with worse survival, but misses some tumors that disguise their aggressiveness with histologic and clinical traits of low risk tumors. We therefore focused our efforts on identifying epigenetic signatures of these deceptive tumors. Several other laboratories have more broadly reported epigenetic features related to poor outcome in ccRCC regardless of tumor risk and SSIGN score, some of which are applicable to biomarker development. For example, Wei et al. [12] developed a five CpG methylation classifier panel using the 450k array on 46 normal-tumor pairs representing a range of stages and grades. The classifier was reproducible in independent cohorts [12]. However, this analysis focused on differences between normal kidney tissue and ccRCCs, which could, by design, fail to capture differences between tumors that distinguish aggressiveness of disease. Indeed, the existence of a set of DNA methylation alterations that characterize ccRCC with poor-outcome is supported by our findings where the altered CpGs specific to our lower risk SSIGN 0–3, STS/LTS cohort also delineate a subgroup of the most aggressive and hypermethylated ccRCCs in a combined TCGA-KIRC and the Swedish cohort irrespective of SSIGN score. Another report showed consistent results with our findings that differentially methylated regions are enriched in kidney H3K4me1-marked regions (poised enhancers). Genes linked to the enhancers were often downregulated and enhancer methylation had prognostic value [21]. Finally, Chen et al.’s [18] work on the interplay between DNA methylation and its oxidation product, hydroxymethylation, showed that 5hmC loss was linked to DNA hypermethylation and that low global 5hmC was associated with poor outcome in ccRCC. It is worth noting that the standard RRBS protocol does not distinguish between DNA methylation and hydroxymethylation, thus some of the ‘hypermethylation’ we observe could result from 5hmC gains. Finally, RRBS, like the 450/850k arrays, interrogate only a modest portion of the total CpG sites in the methylome. As such, additional and/or more robust differential methylation events likely remain to be discovered. Our study would also benefit from being replicated in a larger multicenter cohort of ccRCC samples with SSIGN score 0–3 and by employing machine learning algorithms to develop and test predictive models based on relevant CpGs, as part of future work.

A finding of particular interest from our study was the globally elevated level of DNA methylation in STS, compared to LTS ccRCCs. This difference was even more pronounced at the significant DMCpGs between the two groups, and was consistent across both of our cohorts as measured by RRBS, and at specific loci by pyrosequencing. Although further functional studies are needed to elucidate mechanisms, global hypermethylation may generally promote stemness and/or drive EMT, as suggested by gene ontology analysis. Both of these features are generally associated with more aggressive cancer [36]. Many of the specific loci we identified to be differentially methylated are consistent with this link to EMT. For example, the *PRDM16* gene has functions ranging from regulation of apoptosis, to muscle-brown fat cell fate decisions, hematopoiesis, inflammation, and suppression of EMT [37, 38]. *PTPRN2*, involved in processes ranging from insulin secretion to metastasis and cell migration, is widely reported to be aberrantly methylated in cancer and non-cancer conditions [39, 40]. *SLC12A7* (solute carrier family 12 (potassium/chloride transporter), member 7, or *KCC4*) is normally expressed in the ascending limb of the loop of Henle and is involved in salt reabsorption, however it also functions as a scaffolding protein in the plasma membrane with actin binding protein ezrin. In this context, elevated expression of *SLC12A7* promotes cancer invasion and metastasis through modulation of MMP-2 activity and cell volume control [41, 42]. Regardless of mechanism, the link between global DNA hypermethylation and poor outcome/tumor aggressiveness in ccRCC is a consistent finding across a number of studies. For example, TCGA KIRC linked hypermethylation of 1532 450k probes to poor survival in RCC, and higher disease stage in ccRCC, papillary RCC (pRCC), and chromophobe RCC [17, 18]. Evelönn et al. (2016) observed a hypermethylated ccRCC group associated with higher TNM staging and worse outcome, consistent with an earlier study [43], along with a progressive increase in overall methylation going from normal kidney across stages I-IV (metastatic) of ccRCC [13]. It is also worth noting that none of these previous investigations incorporated SSIGN scores into their survival analyses. We previously showed that *SETD2* mutation in ccRCC was associated with a DNA hypermethylator phenotype, worse patient survival, and greater metastatic potential [11, 14], which was confirmed by TCGA [26]. Taken together, these studies indicate that elevated global DNA methylation in ccRCC is not only a marker of poor outcome, but is also likely a driver of this process. Presumably this is achieved through a combination of tumor suppressor gene hypermethylation and/or silencing of key regulators of EMT and metastasis. The hypermethylator phenotype, however, creates a potential vulnerability of such tumors to DNA hypomethylating agents like 5-aza-2′-deoxycytidine (5-azadC), an FDA-approved epigenetic drug for myeloid disease. Indeed, globally hypermethylated glioma cells (due to *IDH1/2* mutation, another driver of the hypermethylator phenotype) are hypersensitive to DNA hypomethylating drugs [44]. Thus, the most aggressive ccRCCs (including the STS ccRCCs from our study) might also be the tumors most susceptible to drugs like 5-azadC, providing an individualized treatment for patients with hypermethylator tumors. Indeed, the notion of being able to target a subgroup of the most aggressive ccRCCs with epigenetic therapy is bolstered by a recent study that combined DNA methylation and histone deacetylase (HDAC) inhibitors to target lung cancer recurrence and metastasis following surgical resection (which is the front line treatment for ccRCC). Using data from a small group of lung cancer patients, Lu et al. [45] showed that recurrence and metastasis was reduced in patients treated with these agents after resection and was associated with better long-term survival. In mouse models of metastatic lung, breast, and esophageal cancers, adjuvant epigenetic therapy following resection of the primary tumor disrupted the premetastatic microenvironment and inhibited lung metastases, at least in part, by inducing differentiation of myeloid derived suppressor cells [45]. Given that there are no adjuvant therapies in ccRCC that improve overall survival following resection of the primary tumor, coupled with the observation that the most aggressive recurrent ccRCCs tend to be globally hypermethylated, it is intriguing to speculate that this form of epigenetic adjuvant therapy could be particularly well suited to this group of patients most in need of a novel form of treatment. It will be of interest to test such novel strategies for managing ccRCC recurrence in the future.

## Methods

### Clinical samples and clinical/pathological features

Following institutional review board approval, we queried the Mayo Clinic Nephrectomy Registry to identify patients treated with radical nephrectomy for unilateral ccRCC, between the years 1971 and 2010. We identified 1625 cases of which 906 were patients with good prognosis SSIGN scores (0–3 range). After imposing our survival inclusion criteria to identify patients who succumbed to ccRCC in < 6 years (SSIGN 0–1) or < 3.2 years (SSIGN 2–3), which we refer to as short-term survivors (STS), we selected 14 cases for DNA methylation analysis. We also identified a control group of ccRCC patients with SSIGN score 0–3 that remained alive > 10 years (SSIGN 0–1) or > 6.9 years (SSIGN 2–3); referred to here as long-term survivors (LTS), of which we selected 22 cases for DNA methylation analysis. These subjects constituted cohort 1, the discovery cohort. We revisited the nephrectomy registry at a later stage to select a replication cohort (cohort 2) which is made up of 30 LTS and 27 STS patients (57 total). It is important to note that the proportion of LTS and STS tumors in our cohorts is not representative of the proportions in the ccRCC population as a whole, and that cases with SSIGN 0–3 that did not meet our survival inclusion criteria were not considered for this study. LTS and STS groups were frequency matched for SSIGN score, age at surgery, and sex. All pathologic specimens were reviewed by a urologic pathologist blinded to patient outcome for assessment of parameters that go into calculation of the SSIGN score (histologic subtype, tumor size, TNM classification, grade, and necrosis), as described previously [1]. Vital status for patients in the Mayo Clinic Nephrectomy Registry is updated yearly. To establish a normal kidney DNA methylation profile as a reference, we obtained two non-cancerous kidney samples from the Mayo Clinic Biorepository. Detailed clinical information for all samples used in this study is listed in Additional file 1: Table S1 and summarized in Table 1. As a third independent ccRCC patient cohort, publicly available expression (RNA-seq) and DNA methylation (450k array) data were downloaded from TCGA Kidney Clear Cell Carcinoma (KIRC) [6] dataset. To obtain as many samples as possible, we calculated the SSIGN score for these samples and defined short versus long term survivors using more relaxed inclusion criteria than those described above: STS [death in < 7.5 years (SSIGN 0–1) or < 4.5 years (SSIGN 2–3)] and LTS [survival in > 8.5 years (SSIGN 0–1) or > 5.5 years (SSIGN 2–3)]. This resulted in the acquisition of a modestly sized cohort made up of 28 samples: STS (*n* = 9) and LTS (*n* = 19). Furthermore, we included in our study DNA methylation data from a publically available ccRCC cohort on GEO from Sweden (GSE113501) [32], however SSIGN score could not be calculated for these samples due to insufficient clinical/pathological annotation. Instead our analysis was restricted to an ‘all-comers’ ccRCC comparison using TMN stages.

### DNA methylation analysis by RRBS and bisulfite pyrosequencing

Genome-wide DNA methylation was profiled in all samples (normal and ccRCC) through reduced representation bisulfite sequencing (RRBS), as previously described [46, 47]. In brief, after isolation of DNA from FFPE slides for ccRCC samples, or fresh frozen tissue for normal kidney samples, 100 ng of DNA was used for digestion with *MspI*, followed by size selection using standard RRBS protocols to create sequencing libraries. Libraries were sequenced on an Illumina HiSeq2500 at the Mayo Clinic Medical Genome Facility. Quality control and alignment were performed using the SAAP-RRBS pipeline [48]. CpG sites were included in downstream analysis only if they had a coverage depth of at least 5X in ≥ 90% of all ccRCC samples. In cohort 1, the mean number of CpGs covered per sample was 3,243,705 with an overlap of 2,392,937; in cohort 2 the mean number of CpGs was 3,059,894, and the overlap was of 1,153,661.

Select differential methylation events identified by RRBS were confirmed using bisulfite pyrosequencing in 20 ccRCC samples (LTS = 10 and STS = 10; a subset of cohort 1). 500 ng of DNA was bisulfite modified using the EZ DNA Methylation kit (Zymo Research) and sequenced on a PyroMark Q24 (Qiagen) as previously described [49]. PCR and pyrosequencing primers were custom designed using MethPrimer (http://www.urogene.org/methprimer/) (sequences are listed in Additional file 1: Table S8). Sequenced regions did not exceed 100 bp and contained fewer than 10 CpGs. Pyrograms were visualized and methylation levels calculated using Pyromark Q24 v2.0.6 software.

### Published dataset use

To establish an enhancer/promoter landscape for normal kidney, we used publicly available data from Encyclopedia of DNA Elements (ENCODE) for histone marks H3K27ac, H3K4me1, and H3K4me3 with accession numbers GSM1112799, GSM773001, and GSM773005, respectively. These marks were acquired from the kidney of the same 50 year old male. Across the genome, regions with overlapping H3K27ac, H3K4me1, but not H3K4me3, were considered active enhancers. Regions of the genome with H3K4me1 (no H3K27ac and H3K4me3) were considered poised enhancers, whereas regions marked by H3K4me3 were considered promoters. For some figures, promoters were also annotated based on physical proximity to genes using the Bioconductor package *VariantAnnotation* [50].

### Statistical analysis

All analyses were executed in an R environment (version 3.6.2). DNA methylation differences for each CpG site between LTS and STS groups were assessed using independent two-sided *t* tests on beta values, and the criteria for differential methylation were *p* < 0.01 and a change in methylation of 10% (|Δ*β*_(STS-LTS)_|≥ 0.1, as per Yang et al. [51]). Phyloepigenetic trees were constructed using the R packages *ape* and *ggtree,* and heatmaps were constructed using R packages *heatmap3* and *gplots.* Browser views were generated using the *Gviz* package [52]. Ingenuity Pathway Analysis (IPA, Qiagen) and Genomic Regions Enrichment of Annotation Tool (GREAT) [53] were used for gene ontology and comparative analyses. Linear relationships between DNA methylation and gene expression in TCGA data were assessed using Pearson correlation and survival analysis between CpGs in Illumina 450k array and TCGA samples were obtained from the methsurv database [54].

## Supplementary information

**Additional file 1. Table S1**: Detailed summary of RRBS QC parameters and patient data from cohort 1, cohort 2, and normal kidney. **Table S2**: List of differentially methylated CpGs and the gene(s) they are associated with from cohort 1. **Table S3**: Full list of GO biological pathways to which the 5929 DMCpGs were associated using GREAT. **Table S4**: List of kidney-related GO biological pathways to which the 5929 DMCpGs were associated using GREAT. **Table S5**: Full list of canonical pathways to which the 5744 genes obtained from GREAT were associated in IPA. **Table S6**: List of differentially methylated CpGs and the gene(s) they are associated with from cohort 2. **Table S7**: List of 43 CpGs separating aggressive from less aggressive KIRC tumors. **Table S8**: List of PCR and pyrosequencing primers used in this study.

**Additional file 2. Fig. S1**. Global and DMCpG methylation comparison. **Fig. S2**. Unsupervised hierarchical clustering for cohort 1. **Fig. S3** Browser views of the *PTPRN2*, *PRDM16*, *MN1* and *MOB2* genes. **Fig. S4**. Autosomal chromosome ideograms showing locations of DMCpGs for ccRCC cohorts 1 and 2 derived from RRBS. **Fig. S5**. Hexbin plot showing 460 DMCpGs from cohort 2 that are within 1kb of the 5929 DMCpGs from cohort 1. **Fig. S6**. Locus-specific confirmation of RRBS data at three genes (2 regions/gene) using bisulfite pyrosequencing. **Fig. S7**. PCA for the LTS fully methylated (n = 1204) and fully unmethylated (n = 821) CpGs. **Fig. S8**. Supervised hierarchical clustering of KIRC ccRCC samples (n=252) driven by methylation beta values of 22 CpGs (from Fig. 4c) significantly correlated with SSIGN score.

## Data Availability

RRBS-based DNA methylation data generated in this study is available for download in NCBI GEO accession GSE150402.

## Abbreviations

5hmC: 5-Hydroxymethylcytosine
5mC: 5-Methylcytosine
AUA: American Urological Association
ccRCC: Clear cell renal cell carcinoma
DMCpG: Differentially methylated CpG
EMT: Epithelial-to-mesenchymal transition
ENCODE: Encyclopedia of DNA elements
FFPE: Formalin-Fixed Paraffin-Embedded
GREAT: Genomic Regions Enrichment of Annotation Tool
HDAC: Histone deacetylase
HR: Hazard ratio
IPA: Ingenuity Pathway Analysis
LTS: Long term survivor
NCCN: National Comprehensive Cancer Network
pRCC: Papillary renal cell carcinoma
RCC: Renal cell carcinoma
RRBS: Reduced representation bisulfite sequencing
SSIGN: Stage, Size, Grade, and Necrosis
STS: Short term survivor
TCGA-KIRC: The Cancer Genome Atlas Kidney Renal Clear Cell Carcinoma
