# Supplementary material for: Identification of DNA methylation signatures associated with poor outcome in lower-risk Stage, Size, Grade and Necrosis (SSIGN) score clear cell renal cell cancer
Source: Clin Epigenetics. 2021 Jan 18;13:12. doi: 10.1186/s13148-020-00998-z (PMC7814746; doi:10.1186/s13148-020-00998-z)
Supplement: Supplementary file 2 — Additional file 2. Fig. S1. Global and DMCpG methylation comparison. Fig. S2. Unsupervised hierarchical clustering for cohort 1. Fig. S3 Browser views of the PTPRN2, PRDM16, MN1 and MOB2 genes. Fig. S4. Autosomal chromosome ideograms showing locations of DMCpGs for ccRCC cohorts 1 and 2 derived from RRBS. Fig. S5. Hexbin plot showing 460 DMCpGs from cohort 2 that are within 1kb of the 5929 DMCpGs from cohort 1. Fig. S6. Locus-specific confirmation of RRBS data at three genes (2 regions/gene) using bisulfite pyrosequencing. Fig. S7. PCA for the LTS fully methylated (n = 1204) and fully unmethylated (n = 821) CpGs. Fig. S8. Supervised hierarchical clustering of KIRC ccRCC samples (n=252) driven by methylation beta values of 22 CpGs (from Fig. 4c) significantly correlated with SSIGN score. [file 13148_2020_998_MOESM2_ESM.pdf]

Additional File 2: figures and figure legends for 'Identification of DNA methylation signatures associated with poor outcome in lower-risk Stage, Size, Grade and Necrosis (SSIGN) score clear cell renal cell cancer', by El Khoury *et al*.

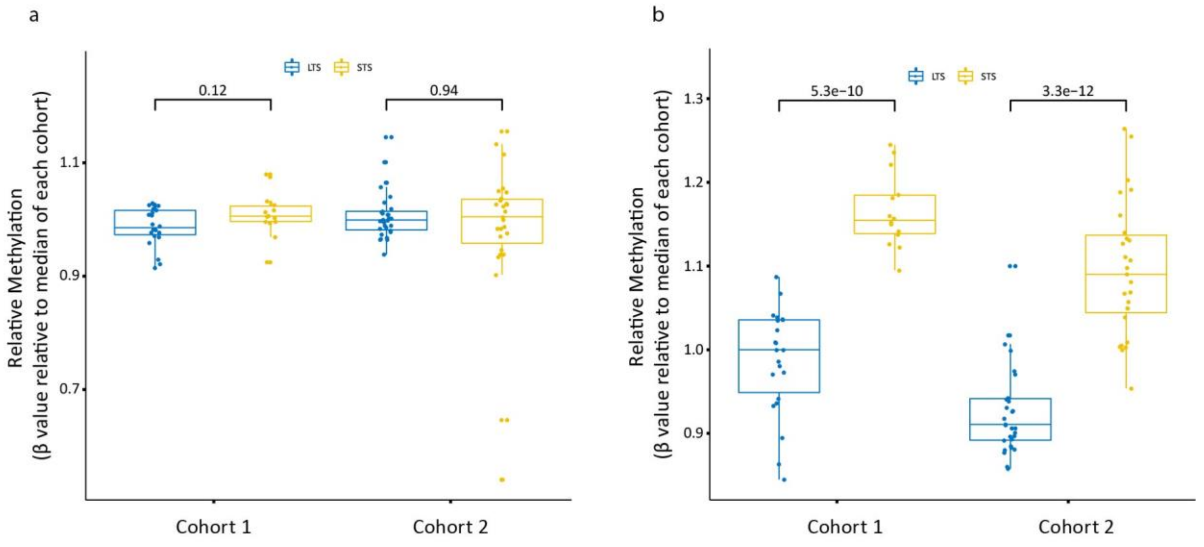

Fig. S1. **a)** Global methylation comparison. In cohort 1 each dot represents the mean methylation of 2,392,937 CpGs for each sample relative to the median of the cohort. In cohort 2 each dot represents the mean methylation of 1,153,661 CpGs for each sample relative to the median of the cohort. Although not statistically significant, STS patients in both cohorts are globally hypermethylated. **b)** Methylation comparison using only the STS versus LTS DMCpGs. In cohort 1 each dot represents the mean methylation of 5,929 DMCpGs for each sample relative to the median of the cohort. In cohort 2 each dot represents the mean methylation of 2,888 DMCpGs for each sample relative to the median of the cohort.

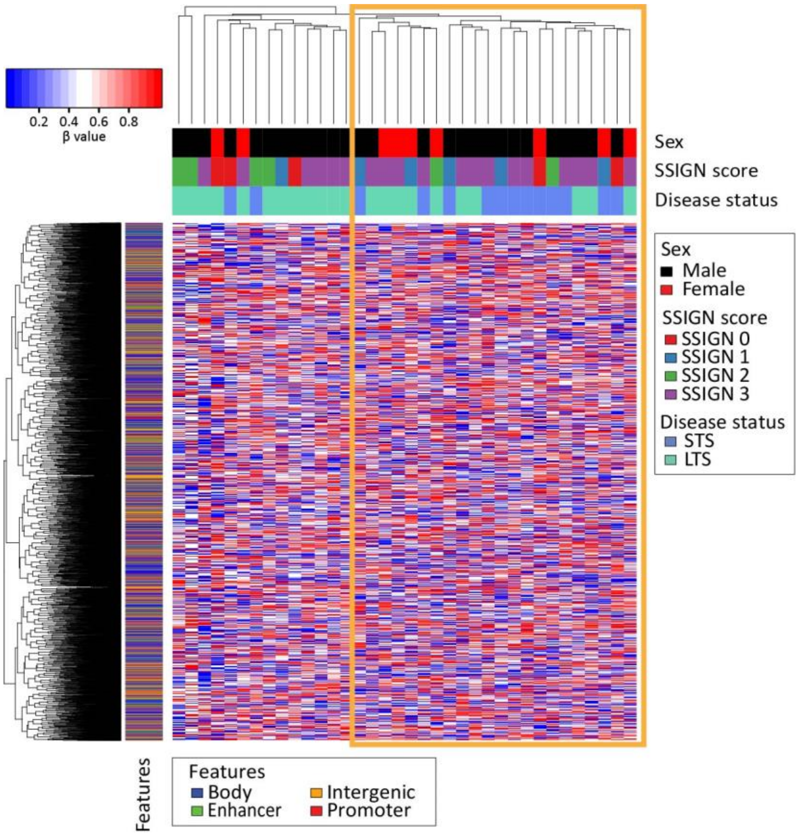

Fig. S2. Heatmap showing unsupervised hierarchical clustering of cohort 1 (n=36) driven by methylation beta values at the 5,929 most variable CpGs. Color bars beneath the column dendrogram represent, from top to bottom: sex, SSIGN score, and disease status. We observe a significant over-representation (p = 0.016) of STS samples on the right side of the dendrogram (orange box).

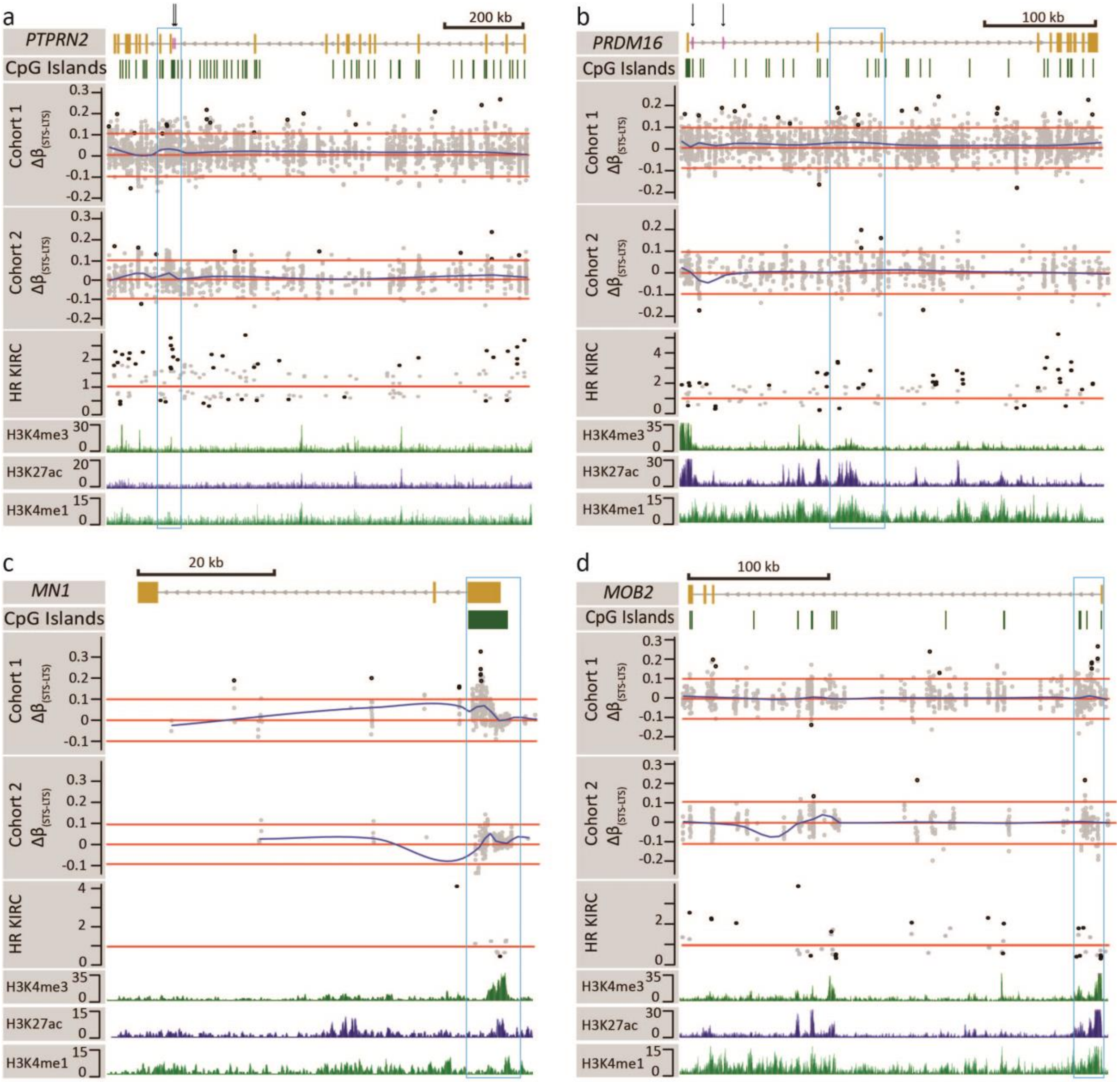

Fig. S3. a-d) Browser views of the *PTPRN2, PRDM16, MN1* and *MOB2* genes. Genes of interest are displayed in the first track. Yellow bars represent exons and the grey line represents introns. Black arrows pointing to short purple bars represent the location of loci that underwent pyrosequencing (a and b only). The direction of transcription is indicated by the arrows on the grey line. The second track displays CpG islands (green bars). Tracks 3 and 4 represent the $\Delta\beta_{(STS - LTS)}$ of all CpGs covered by RRBS 5,000 bp upstream and downstream of the gene in cohorts 1 and 2, respectively (data for cohort 1 is also shown in Fig. 2 for some loci). Each grey circle represents a CpG, and the black circles represent

DMCpGs meeting $p<0.01$ and $|\Delta\beta_{(STS - LTS)}|>0.1$ cutoff. Red lines represent the cutoff line at $\Delta\beta_{(STS - LTS)}$ ±0.1. The blue line is a smoothed distribution of the data. Track 4 depicts the hazard ratio of CpGs derived from the Illumina 450K array and measured in TCGA-KIRC that are in the vicinity of DMCpGs identified by RRBS. Each circle represents a CpG, and those in black are significantly associated with survival in KIRC. CpGs with HR>1 are associated with poor survival when hypermethylated. CpGs with HR<1 are associated with better survival when hypermethylated. Tracks 5-7 display ChIP-seq data from normal kidney for the histone marks H3K4me3, H3K27ac, and H3K4me1, respectively. Blue vertical rectangles highlight regions of interest where CpGs with regulatory roles can be located.

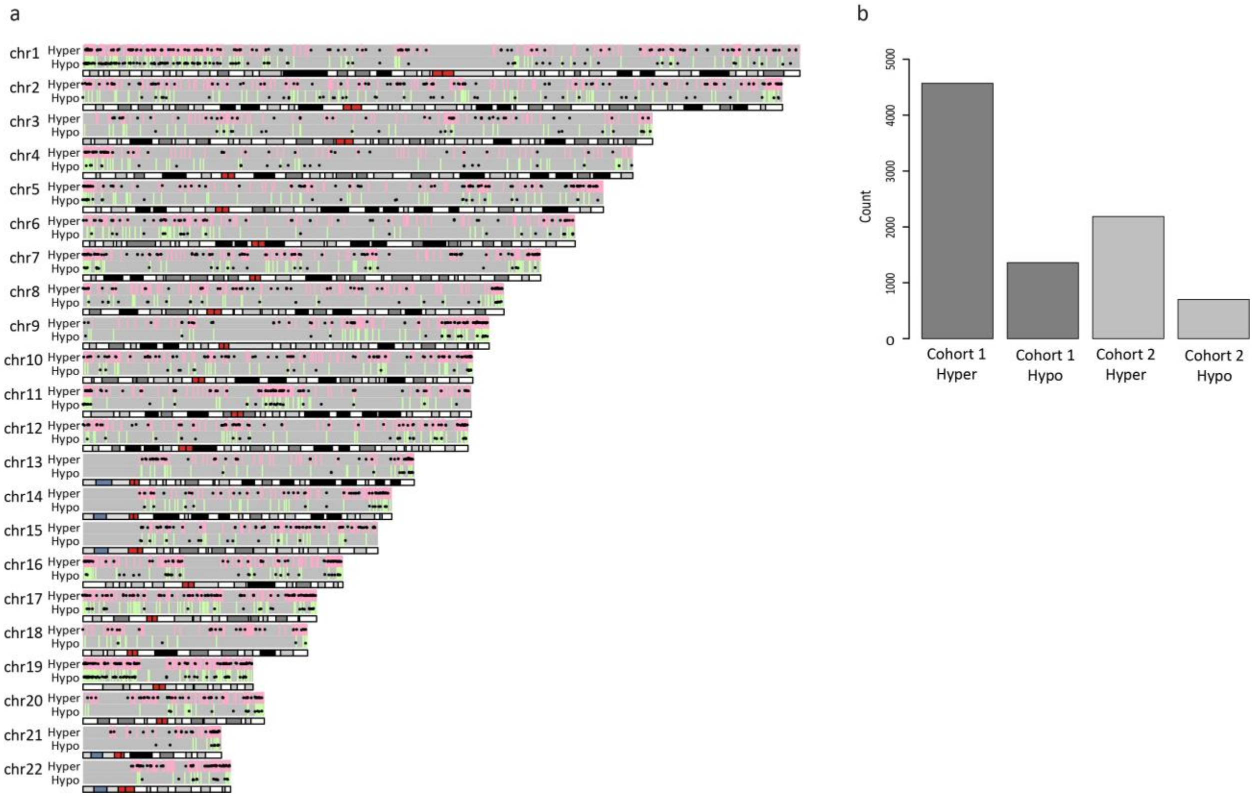

Fig. S4. **a)** Autosomal chromosome ideograms showing locations of DMCpGs for ccRCC cohorts 1 and 2 derived from RRBS. Pink bars (top of each chromosome) represent the location of hypermethylated DMCpGs from cohort 1 (n=4,570). The black dots in the 'hyper' panel for each chromosome represent hypermethylated DMCpGs from cohort 2 (n=2,186). Green bars represent the location of hypomethylated DMCpGs from cohort 1 (n=1,359). The black dots in the 'hypo' panel represent hypomethylated DMCpGs from cohort 2 (n=702). **b)** Barplot showing the number of hypermethylated and hypomethylated DMCpGs from cohorts 1 and 2.

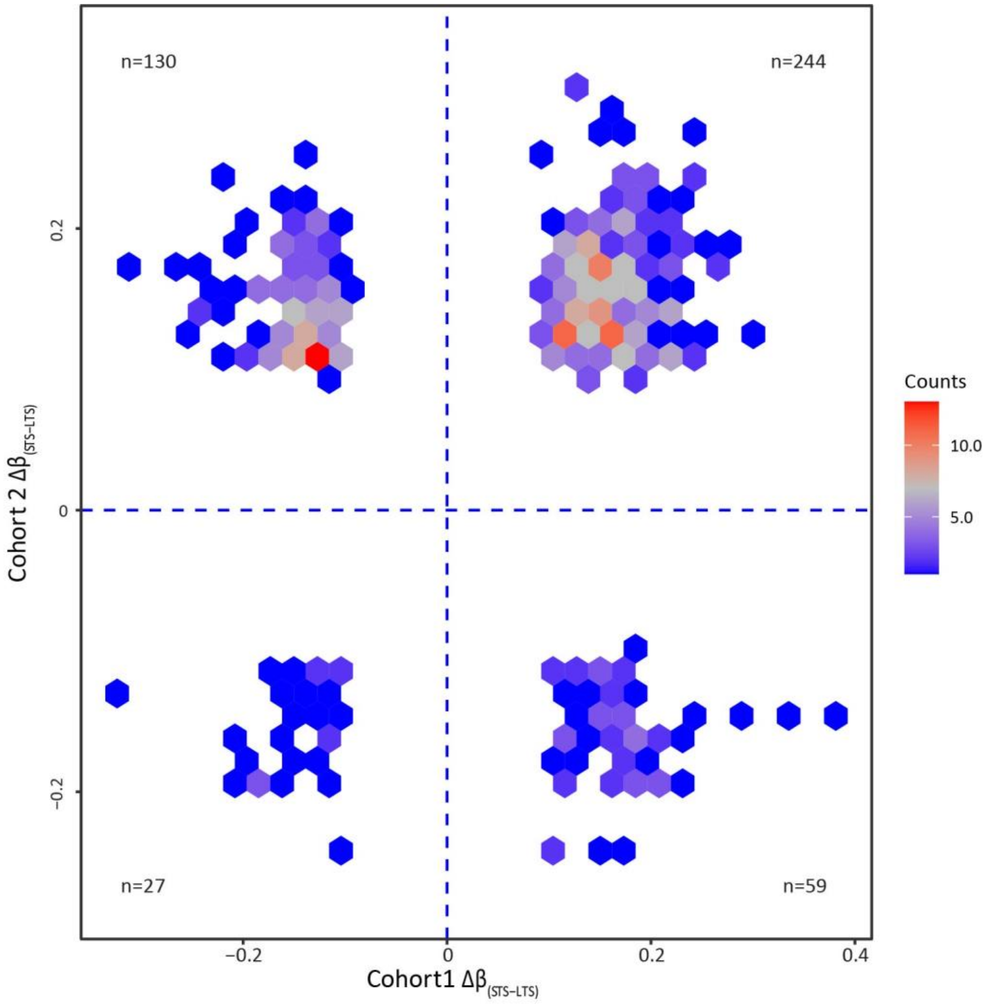

Fig. S5. Hexbin plot showing 460 DMCpGs from cohort 2 that are within 1kb of the 5,929 DMCpGs ($|\Delta\beta|>0.1$) from cohort 1. Numbers in each corner represent the number of CpGs in the respective quadrant. We observe a greater number of convergent than divergent changes in the directionality of methylation in STS relative to LTS for both cohorts.

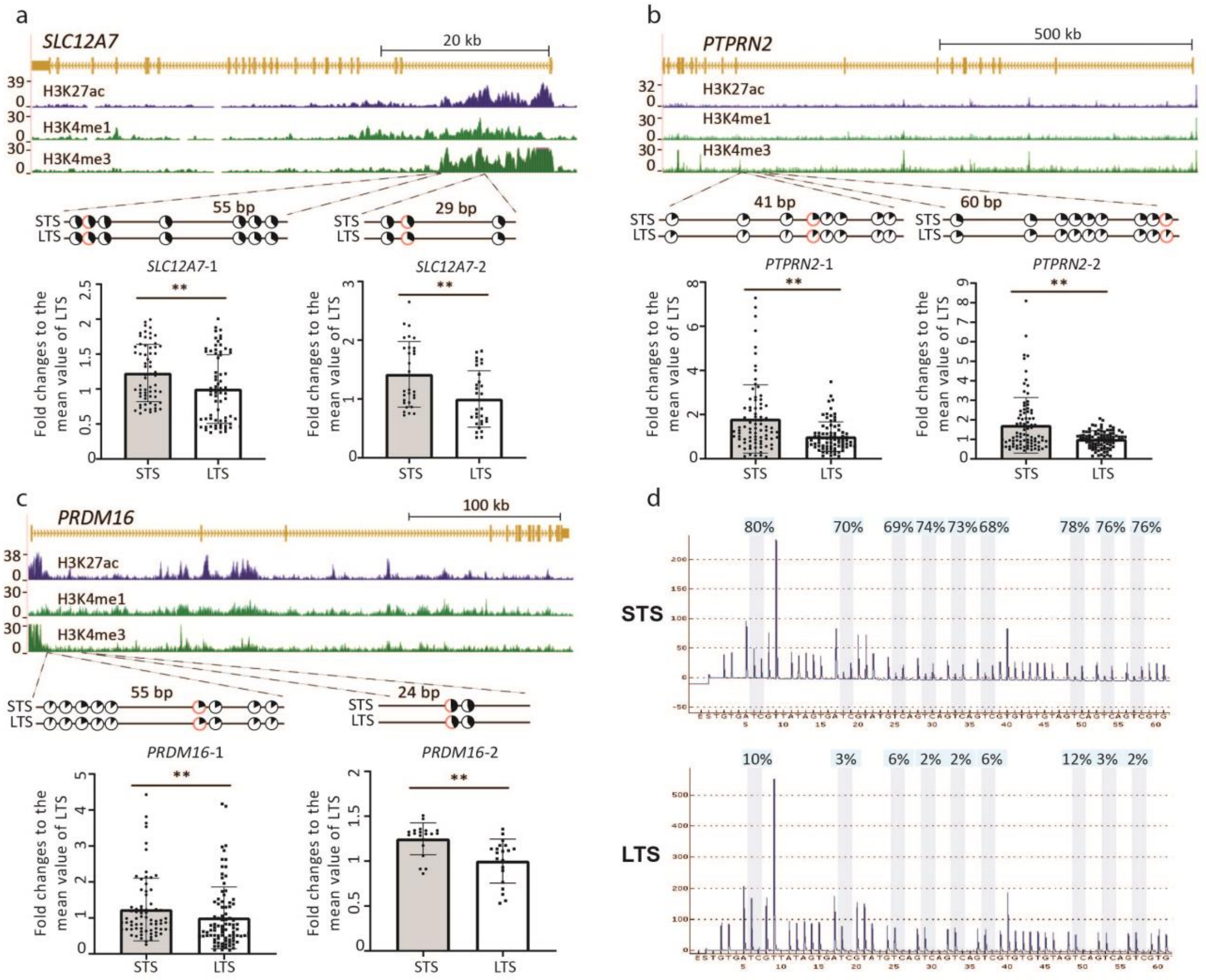

Fig. S6. Locus-specific confirmation of RRBS data at three genes (2 regions/gene) using bisulfite pyrosequencing. Panels a-c summarize pyrosequencing data for the **a)** *SLC12A7*, **b)** *PTPRN2*, and **c)** *PRDM16* genes. Each panel is composed of a browser view spanning 3 kb upstream of the transcription starting site (TSS) and downstream of the last base pair of the gene. Browser views show the gene structure (introns yellow/orange lines, exons yellow/orange bars), and histone ChIP-seq tracks for H3K27ac, H3K4me1, and H3K4me3 derived from normal kidney. Each panel also contains pie charts representing CpGs covered by pyrosequencing primers. The black coloring of each pie represents the mean methylation level for STS (n=10) and LTS (n=10) samples from cohort 1. Pies with a red outline represent DMCpGs identified in cohort 1. Finally, each panel contains vertical scatter-barplots

summarizing the relative difference between LTS and STS for each surveyed CpG: *SLC12A7*-1 (STS n=63, LTS n=70), *SLC12A7*-2 (STS n=30, LTS n=30), *PTPRN2*-1 (STS n=80, LTS n=80), *PTPRN2*-2 (STS n=90, LTS n=90), *PRDM16*-1 (STS n=72, LTS n=90), and *PRDM16*-2 (STS n=20, LTS n=20). Each dot represents a CpG in every studied sample. The Y-axis represents the relative difference between the methylation level of each CpG per sample and the median methylation of the LTS group. Error bars represent standard deviation. Two asterisks (**) represent Mann Whitney U test p<0.01. **d)** Representative pyrograms of *PTPRN2* region 2 as displayed on the pyromark software. Regions with grey shadows represent the location of a CpG. The X-axis is the dispensation sequence. The Y-axis indicates light intensity emitted when nucleotides anneal to the newly generated sequence. The height of the peak is indicative of repeats of the same nucleotide in the genetic sequence.

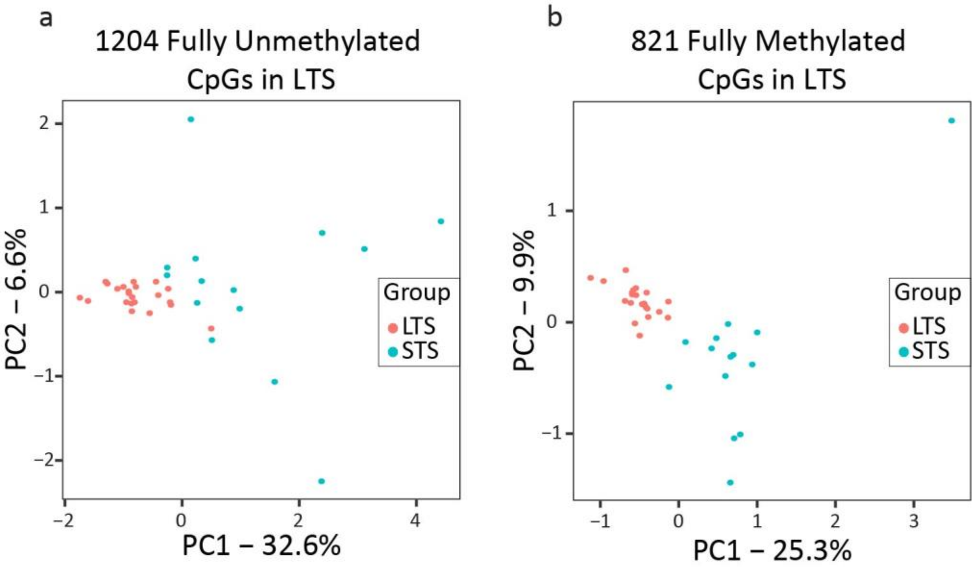

Fig. S7. PCA for the **a)** LTS fully methylated (n=1204) and **b)** fully unmethylated (n=1380) CpGs demonstrating separation between LTS (red) and STS (blue) groups (compare with Fig. 4e). The percentages at each axis represent the variance explained by each respective principal component.

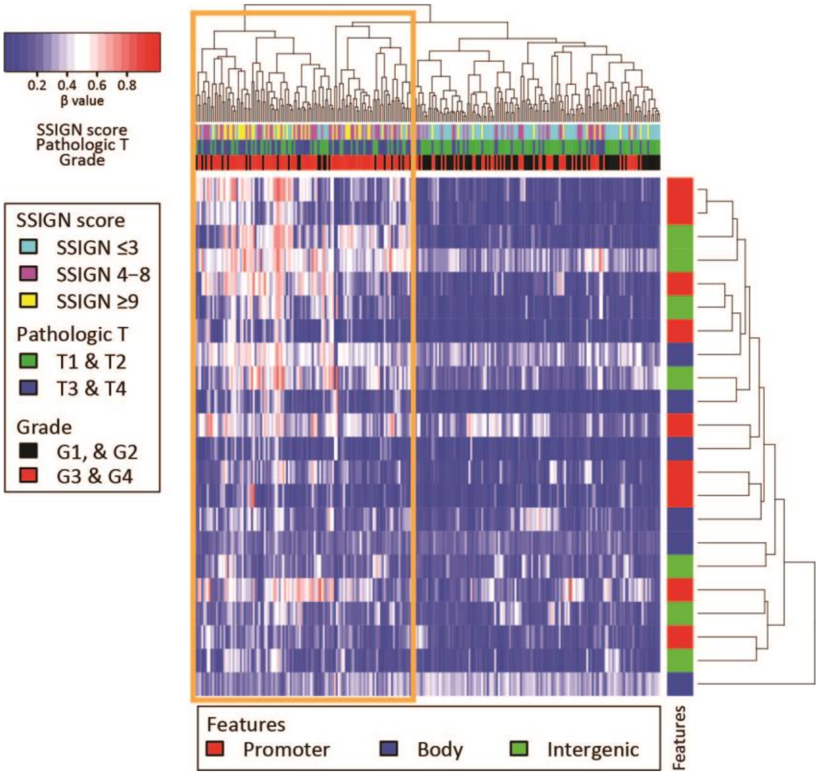

Fig. S8. Heatmap showing supervised hierarchical clustering of KIRC ccRCC samples (n=252) driven by methylation beta values of 22 CpGs significantly correlated with SSIGN score. Color bars beneath the column dendrogram represent, from top to bottom: SSIGN score, pathologic T stage, and cancer grade. SSIGN scores are categorically divided into 3 groups: ≤3, 4-8, and ≥9. Pathologic T stages are divided into low (T1 and T2) and high (T3 and T4) stages. Cancer grades are divided into low (G1, and G2), and high (G3 and G4). The color bar next to the row dendrogram indicates the genomic features. When examining 22 CpGs aggressive tumors cluster into one distinct group (orange box).
